# Analyzing Kernel Matrices for the Identification of Differentially Expressed Genes

**DOI:** 10.1371/journal.pone.0081683

**Published:** 2013-12-09

**Authors:** Xiao-Lei Xia, Huanlai Xing, Xueqin Liu

**Affiliations:** 1 School of Mechanical and Electrical Engineering, Jiaxing University, Jiaxing, P.R. China; 2 School of Information Science and Technology, Southwest Jiaotong University, Chengdu, P.R. China; 3 School of Electronics, Electrical Engineering and Computer Science, Queen's University Belfast, Belfast, United Kingdom; Queen's University Belfast, United Kingdom

## Abstract

One of the most important applications of microarray data is the class prediction of biological samples. For this purpose, statistical tests have often been applied to identify the differentially expressed genes (DEGs), followed by the employment of the state-of-the-art learning machines including the Support Vector Machines (SVM) in particular. The SVM is a typical sample-based classifier whose performance comes down to how discriminant samples are. However, DEGs identified by statistical tests are not guaranteed to result in a training dataset composed of discriminant samples. To tackle this problem, a novel gene ranking method namely the Kernel Matrix Gene Selection (KMGS) is proposed. The rationale of the method, which roots in the fundamental ideas of the SVM algorithm, is described. The notion of ''the separability of a sample'' which is estimated by performing 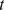-like statistics on each column of the kernel matrix, is first introduced. The separability of a classification problem is then measured, from which the significance of a specific gene is deduced. Also described is a method of Kernel Matrix Sequential Forward Selection (KMSFS) which shares the KMGS method's essential ideas but proceeds in a greedy manner. On three public microarray datasets, our proposed algorithms achieved noticeably competitive performance in terms of the B.632+ error rate.

## Introduction

Microarray data has been applied to the class prediction of different samples, from which the disease diagnosis and prognosis can benefit. A microarray dataset usually contains thousand of genes and a relatively much smaller number of samples (usually 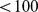). For the purpose of predicting the type of biological samples, a majority of this genes are irrelevant and redundant. This fact has prompted the development of a variety of approaches which detect differentially expressed genes (DEGs) to accomplish an accurate classification of the samples.

The 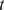-test has been one of the most widely-used parametric statistical methods for the identification of DEGs between populations of two classes. Variants of the 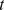-test, which adopt different technologies to obtain a more stable estimate of the within-class variance for each gene, have been proposed [1]–[3]. The regularized 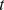-test, for example, adjusted the gene-wise variance estimate by using a Bayesian probabilistic model [2]. For multiple testings, the 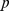-value is calculated and adjusted to address the problem that the false positive rate is likely to accumulate over thousands of genes. Approaches in this categories range from those bounding the ''Family-Wise Error Rate'' (FWER) which is the overall chance of one or more false positives [4]–[6] and strategies controlling the ''False Discovery Rate'' (FDR) which is the expected percentage of false positives among the genes deemed as differentially expressed [1], [7]. Because the null distribution is unknown, these methods often shuffle the class labels of the samples to estimate the 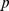-value. The ANOVA 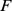-test extends the 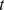-test to multiple classes and a number of 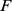-like statistics have been proposed which used different shrinkage estimators of the gene-wise variance [8], [9].

Another family of statistical methods proposed to factor in the dependency information between genes. Representative examples include the gene pair selection method [10] and correlation-based methods the rationale behind which is that a good feature subset is highly correlated with the class and uncorrelated with each other [11], [12]. Also included are the approaches derived from Markov blanket filtering [13]–[15]. Minimum redundancy maximum relevance [16] and uncorrelated shrunken centroid [17] are also well-established gene selection methods in this category.

When cast in the framework of pattern recognition, gene selection is a typical feature selection problem. Feature selection techniques in pattern recognition can be generalized into three types: filter, wrapper and embedded methods [18]–[20]. For filter methods, the feature selection is performed independently of a classification algorithm, which cover a majority of the aforementioned statistical tests. Wrapper methods, by contrast, use a classifier to evaluate a feature subset. The problem of choosing 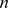 out of 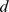 features involves altogether 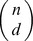 feature subsets. An exhaustive evaluation of these subsets is computationally infeasible, particularly for microarray data of a large 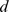. A number of heuristic search techniques are thus proposed, and among them are the Sequential Forward Selection (SFS), the Sequential Backward Elimination (SBE), the Sequential Forward Floating Selection (SFFS) and the Sequential Backward Floating Elimination (SBFE). The SFS has been used to search for feature subsets which are evaluated by the leave-one-out cross validation accuracy of Least-Squares SVM [21], [22]. Genetic Algorithms (GAs) are another family of search strategies that have attracted considerable research attention [23]–[25].

Embedded methods, on the other hand, use the intrinsic property of a specific classifier to evaluate feature subsets. For example, the SVM Recursive Feature Elimination (SVM-RFE) [26] regards that the normal vector of the linear SVM carries the significance information of the genes. Representative examples also include random forest induced approaches [27], [28]. An extensive review of major feature selection techniques has been carried out [29]. No general consensus has yet been reached on which one is the best, despite the diversity and abundance of gene selection algorithms.

Empirically, wrappers and embedded methods have been observed to be more accurate than filters [30]. However, they require repetitive training of a specific classifier in order to guide the search in the space of feature subsets and are consequently very time consuming. Filters are, generally speaking, faster in the absence of interactions between feature subsets and a classifier. Thus filters, statistical tests in particular, have enjoyed considerable popularity in the field of gene selection for microarray data [4], [9], [31], [32]. In fact, wrappers normally incorporate statistical tests as a preprocessing step to prune a majority of genes so that the number of feature subsets to be visited is reduced along the search pathway [21], [22], [26].

Meanwhile, although the choice of the classifier also presents a wide diversity, SVMs have been widely recognized for its generalization abilities [33] and remained as a predominant option [34]–[36].

In summary, a widely-accepted scheme for the analysis of microarray data has been ''identification of DEGs by statistical tests followed by sample classification using SVMs''. The justification is that the prediction accuracy of various classifiers including SVMs, depends on how discriminant the features are. However, SVMs belong to the family of sample-based classifiers whose generalization performance comes down to, more precisely, how discriminant the samples are. DEGs identified by statistical tests cannot guaranteed to establish a set of discriminant samples for SVMs. Consequently, it cannot be promised the highest degree of accuracy for sample classification. This problem necessitates the development of gene selection algorithms that are more consistent with the fundamental ideas of SVMs. It is naturally desired that, the proposed methods can bypass the computationally-expensive training procedure of SVMs, which is required by the SVM-RFE algorithm [26] and wrapper methods based on Least-Squares SVMs [21], [22].

## Materials and Methods

### Support Vector Machines

Given a binary classification problem with the training data set of: 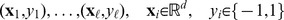
(1)where 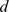 is the number of features and each 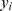 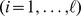 is the class label for the training sample 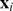.

**Figure 1:**
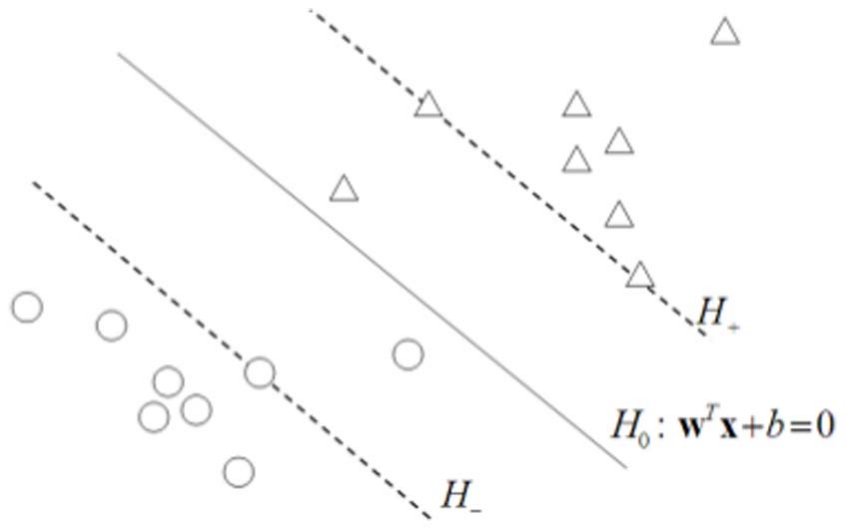
The linear SVM trained on samples from two classes. Samples locating on the hyperplanes of 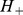 and 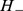 are referred to as ''boundary samples''.

As depicted in Fig. 1, the SVM algorithm seeks the separating hyperplane 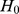 which possesses optimal generalization abilities. The hyperplane 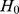 takes the form of 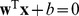 where 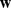 is the normal vector and the constant 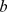 the bias term. The classifier 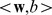 is constructed so that samples from the positive class lie above the hyperplane 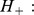 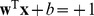 while samples from the negative class lie beneath the hyperplane 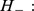 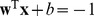.

The condition of optimality requires that the vector 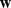 be a linear combination of the training samples: 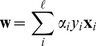
(2)


Each constant 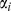 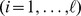 is the Lagrangian multiplier introduced for sample 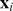. The feasible value range for the 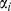
's is 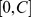 where 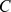 is the regularization parameter and tunes the tradeoff between generalization abilities and the empirical risk.

For nonlinear problems where the training data are not separable in the input space, a function, denoted as 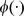, is applied, mapping the data to a feature space of higher dimensions where they become separable. Consequently, the normal vector of the resultant classifier becomes: 
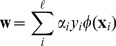
(3)


Equation (2) which represents the solution in the linear case, can also be viewed as a special case of Equation (3) where 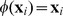.

On a test sample 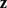, the SVM classifier outputs a decision value of: 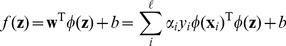
(4)


According to the sign of the 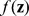, the sample 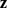 obtains a class label of either 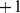 or 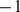.

Equation (4) suggests that the SVM algorithm requires the knowledge of the dot product between 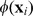, rather than that of 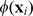 itself. Thus the SVM employs the ''kernel trick'' which allows the the dot product between 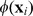 to be computed without the explicit knowledge of the function 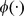.

### Mining the Information Hidden in the SVM Solution

As mentioned previously, each training sample is eventually assigned a Lagrangian multiplier 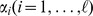, subject to 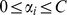. The establishment of the SVM classifier is, in actual fact, a process of optimizing the values of these 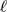 Lagrangian multipliers. In the SVM solution which is formulated by Equation (4), 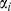
's can be divided into three groups which respectively satisfy 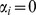, 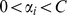 and 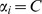.

Using Figure 1, we now focus on the linear SVM classifier and review the connection between the value of 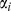 and the geometric location of its associated training sample 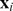. It is worth attention that the connection arises, mathematically, from the optimality conditions of SVMs [37], [38]. We then reveal the hidden information that can be mined out of this connection.

1. 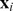 with 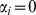


Depending on its class label 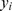, 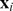 lies geometrically either in the space above 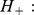 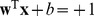 for 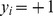 or in the space beneath 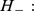 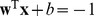 for 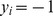.

Consider a sample 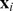 whose 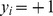. Since it locates in the subspace above 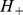, we expect 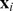 bearing noticeable similarities to class ''+'' than to class ''
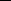
''. The similarity of 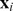 and class ''+'' can be measured by evaluating the the similarity between 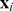 and each representative sample from class ''+''. The training set of the SVM is, or has been supposed to be, composed of representative samples from each class.

We use the inner product to measure the similarity level between vectors. Denoting the number of the positive training samples as 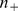, the inner products between 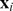 and each each positive training sample 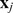 form a population of 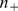 measurements, denoted as 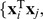 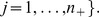 The mean of these measurements, denoted as 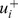, is regarded to be indicative of the similarity of 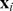 and class ''+'': 
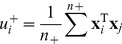
(5)


Likewise, denoting the number of the negative training samples as 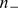, the similarity of 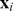 and class ''
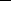
'' can be measured as: 
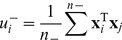
(6)where the set 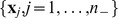 consists of all the negative training samples.

As a result, we can express, mathematically, the expectation that a positive sample bears more resemblance to class ''+'' than to class ''
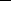
'' as: 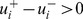
(7)


And a negative training sample 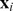 whose 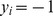 and 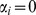 is expected to satisfy: 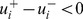
(8)


Equation (7) and Equation (8) can be combined into: 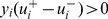
(9)


2. 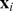 with 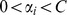



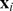
 with 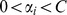 lies exactly on either 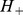 for 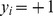 or 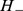 for 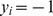. This group of training samples are normally referred to as ''boundary samples''.

The class resemblance of a boundary sample to its supposed class is not as striking as those samples with 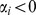. Nevertheless, they are still the samples whose class labels can be correctly restored by the SVM solution and thus expected to satisfy Equation (9).

3. 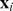 with 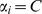



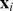
 whose 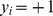 and 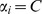 can be located at one of the following three locations:

(a) exactly on the hyperplane of 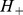;
(b) in the region between the hyperplanes of 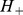 and 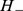 but closer to 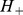;
(c) in the region between the hyperplanes of 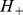 and 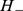 but closer to 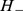.

A training sample from group (a), is a boundary sample but its class label can be correctly restored by the SVM solution. As with positive samples whose 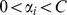, Equation (9) is expected to hold for samples from case (a).

For a training sample from group (b), the SVM classifier would not have been able to correctly restore its class label if it weren't for the introduction of the slack variables. Our interpretation is that, the class resemblance of this sample to its supposed class is so ambiguous that the SVM has difficulties in acknowledging its actual class membership.

For a training sample from group (c), the SVM classifier is simply unable to correctly restore its class label. It is very likely that the class resemblance of this sample to its supposed class in fact contradicts its given class label.

In mathematical terms, we reckon that a positive training sample of either group (b) or group (c) satisfies: 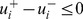
(10)


The hidden information for 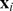 whose 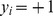 and 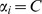 can be inferred in a similar manner. And the formulation that describes a sample 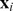 with 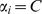 can be generalized as: 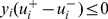
(11)


In summary, the resultant value of 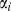 for the training samples 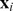 suggests how discriminant 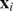 is between two opposing classes. But the values of 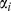
's can only be obtained after the completion of the training procedure which is of a formidable time complexity of 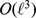.

Luckily, our analysis above implies that that the function of 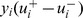 is, promisingly, indicative of the discriminant level of 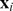. In other words, the vector of 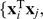 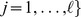 is highly informative about the complexity of classifying 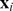 by the linear SVM classifier. It is easy to infer that, for nonlinear problems, this information can be obtained from the vector of 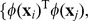 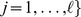.

### Estimating the Separability of a Problem

In the SVM algorithm, the vector of 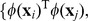 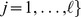 constitutes the 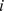-th column of the input kernel matrix. The 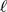 measurements in the 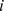-th column can be separated into two populations, according to the class label of sample 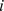, and respectively denoted as 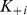 and 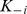. Performing the following test to the two populations yields a score 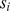 which measures the separability of 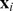: 
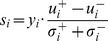
(12)where 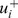
(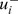) and 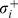
(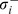) are the mean and the standard deviation of 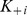 (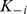).

We justify the introduction of standard deviations in the denominator by considering two positive training samples in the feature space. The first sample is assumed to have come from a region of denser population than the second one. We reckon that, compared with the second sample, the first sample is more typical a representative of class ''+'' and is believed to be more similar to class ''+''. The positive sample from a denser population is expected to exhibit a lower deviation of the elements 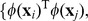 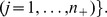 Thus, the standard deviation is formulated into Equation (12), demonstrating our confidence in a higher separability of a sample from a denser population.

The values of 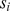
's can be split into three types, large positive ones, small positive ones and negative ones. A large positive value of 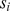 implies that, the training sample 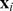 is likely to be discriminant, statistically bearing more resemblance to the supposed class than to the other one. A small positive 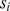 suggests that, 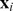 might bear almost the same level of resemblance to both classes and thus, hard to classify. For a negative value on 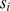, the class that 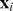 is computed as more similar to, is different from the actual one, which poses difficulties for the SVM classifier.

Meanwhile, the similarity between each sample and itself is supposed to be 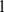. However, It is not the case for all kernel functions to satisfy 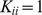. Consequently a proper preprocessing procedure might be required prior to the application of Equation (12), depending on the kernel in use. For linear kernels, we divide each element of the 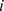-th column of the kernel matrix by 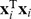. For Gaussian RBF kernels [29] which take the form of 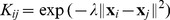
(13)it already holds that 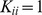 and the preprocessing step is avoided. However, the value of the parameter 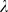 is required to be optimized.

Since the separability of each sample has an impact on the the class separability of a problem, we propose to use the sum of each sample's separability score as an estimate of the separability of the problem.

A word about the formulation of Equation (12). In statistics, it is the norm of practice to add a small constant to the sum of variances, in order to guard against zero in the denominator. But for our algorithms, the designation of 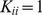 prevents the occurrence of zero in the denominator of Equation (12). We explain how it is achieved for linear kernels and Gaussian RBF kernels:

1. In the case of linear kernels, take a positive sample 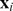 for example. Since 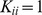, in order to have 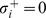 for Equation (12), it demands that 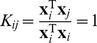 for any 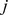 whose 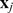 is a positive sample. This requires that 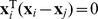. This set of conditions can only satisfied either when 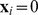 or 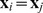 which suggests that the training set only include one positive sample. We reckon that either case is unlikely for well-posed classification problems.
2. In the case of Gaussian RBF kernels, in order to have 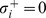 given a positive sample, it has to be met that 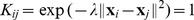 for any 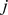 whose 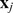 is a positive sample. This in fact implies that either 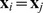 which is hardly true with real-life microarray datasets, or the parameter 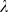 has been assigned a value of zero, which can be easily avoided.

### Kernel Matrix Induced Gene Selection Algorithms

**Figure 2:**
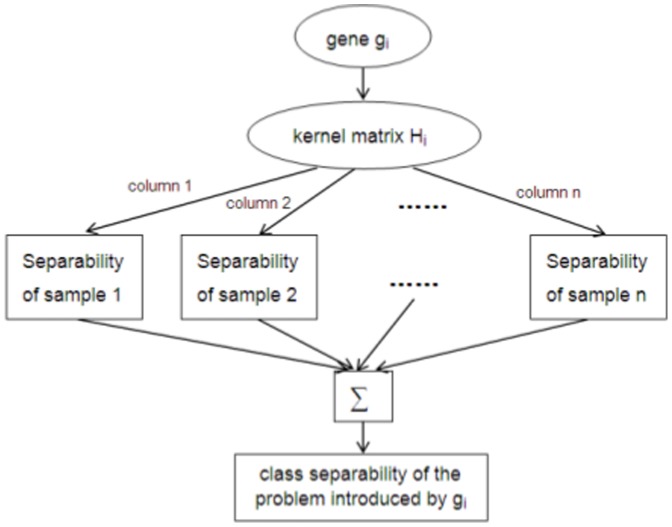
The essential idea of kernel matrix induced gene selection algorithms.

Since each gene subset introduces a classification problem represented by the set of training samples, the gene subset thus corresponds to an estimate of the separability of the problem. Consequently, DEGs can be identified as those resulting in ''easier problems'' of high separability. This is the essential idea of our kernel matrix induced gene selection methods, which has been illustrated in Figure 2. This methodology is shared by the two gene selection algorithms we proposed below. The first algorithm, namely the Kernel Matrix Gene Selection (KMGS), ranks each gene individually, while the second one, namely the Kernel Matrix Sequential Forward Selection (KMSFS), identifies DEGs iteratively.

#### Kernel Matrix Gene Selection

Given a microarray dataset of 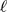 samples with 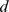 genes, the 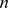-th 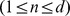 gene of the 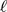 samples forms a vector. The vector, in fact, establishes a training set for the following classification problem: 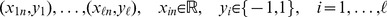
(14)where 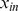 is the value of 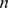-the gene for the 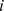-th sample and the 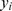 is its given class label. Given the training set, the separability of each sample, denoted as 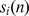, can be assessed using Equation (12). The class separability of the problem constructed from the 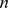-th gene can thus be computed:
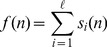
(15)while the reason behind using (15) is that the class separability of a problem is reflected by the sum of the separability of each sample.

Hence the function 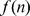 maps a gene to the separability level, in the contexts of sample-based classifiers including the SVM.

The 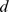 genes are ranked according to their respective 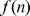 value where 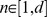. Genes achieving a large 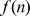 obtain higher rankings.

#### Kernel Matrix Sequential Forward Selection

An alternative to the KMGS which proceeds in a greedy manner is also developed, which is namely the Kernel Matrix Sequential Forward Selection (KMSFS) algorithm. The algorithm starts with an empty set of selected DEGs. At each iteration, the algorithm identifies a single DEG which is then appended to the set. We now describe how the KMSFS algorithm proceeds between two consecutive iterations.

Given a microarray dataset of 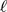 samples with 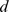 genes, at the 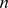-th iteration, 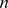 genes has been collected into the set of DEGs. This in fact stands for a classification problem with the training set composed of 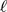 samples, each of which is of 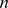 dimensions: 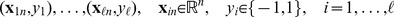
(16)


Each gene from the remaining 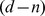 genes is, in turn, appended to these 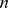 genes and forms a different classification problem with a training set of 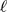 samples, each of which is of 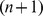 dimensions. This results in, altogether,  data matrices of size 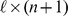 which are actually the training sets for 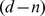 classification problems. The complexity of each problem can be estimated and interpreted as the significance of the associated 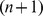-th gene. The 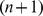-th DEG is eventually identified to be the one which produces the problem featuring the highest separability.

The pseudo codes for the KMGS and KMSFS algorithms are given respectively in Table 1 and Table 2.

**Table 1:** The Algorithm of Kernel Matrix Gene Selection.

INPUT:	
	- The data set 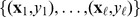 where 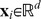 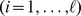
	- 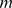 is a user-defined integer which indicates the number of selected genes.
FOR 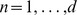	
	- A training data set is formed by the 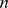 -th feature of the original training set:
	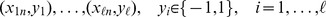
	where 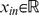 is the 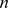 -th feature of the 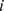 -th sample 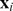 .
	- Construct the kernel matrix 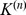 . In the case of the linear kernel function, apply the aforementioned preprocessing procedure to ensure that: 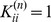 for each column.
	- At the 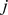 -th column entry, the measurement 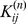 is assigned into either the population 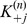 or 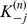 depending on the class label 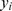 . Perform the following statistical test on the two populations:
	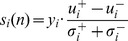
	where 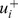 ( 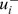 ) and 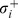 ( 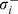 ) are the means and standard deviations of 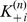 ( 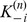 ).
	- The class separability w.r.t feature 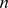 can be assessed by 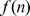 :
	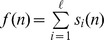
OUTPUT:	
	- Choose the genes which correspond to the top 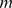 values of 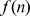 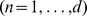

**Table 2:** The Algorithm of Kernel Matrix Sequential Forward Selection.

INPUT:	
	- The data set 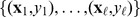 where 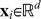 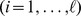
	- 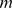 is a user-defined integer which indicates the number of selected genes.
	- γ is the index set of candidate features. Initially 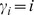 where 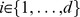 .
	- 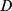 is a 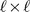 matrix which is 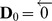 at the start.
-FOR 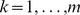	
	- FOR 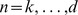
	- A matrix 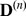 is established whose element 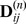 is:
	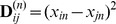
	where 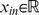 is the 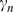 -th feature of the original 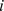 -th training sample 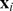 .
	- Add the 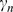 -th feature to the set of previously 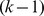 selected features whose indices are 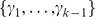 .
	A training data set is formed by the 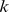 features of the original training set:
	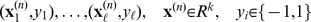
	- Construct the kernel matrix 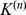 . In the case of the linear kernel function, apply the aforementioned preprocessing procedure to ensure that 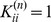 for each column.
	- Calculate the score 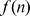 w.r.t 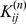 using (12) and (15).
	- The 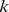 -th iteration of the outer loop finds a feature 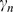 :
	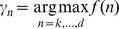
	- Swap 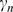 and 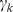 so that 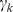 always points to the newly selected feature:
	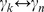
	- Update 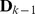 to 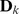 :
	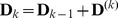
OUTPUT:	
	- The index of 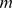 selected genes are given by 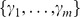 in chronological order.

## Merits of Proposed Algorithms

The proposed methods have noticeable merits:

1. Filter methods identify discriminant features, making them suitable for feature-based classifiers whose normal vector is the linear combination of features. However, Equation (3) demonstrates that the SVM classifier is the linear combination of training samples in the feature space. Thus the performance of the SVM comes down, more to discriminant levels of samples than those of features. Since discriminant features selected by filter methods are not guaranteed to generate a training set composed of discriminant samples, the resultant classifier cannot be ensured to be optimally accurate either. In contrast, our algorithms aim at unveiling the information regarding discriminant levels of samples using the kernel function. Our algorithms are developed upon the fundamental ideas of SVMs and thus more likely to produce a classifier of a higher degree of accuracy.
2. A majority of wrapper and embedded methods are based on the assumption that most microarray datasets pose linear problems. However, we reckon that, the problem presented by a set of DEGs can hardly be a linear one when the the set size is as small as only one or two.

But the generalization to nonlinear cases have been challenging for various wrappers and embedded methods. For example, the SVM-RFE [27] keeps unchanged the Lagrangian multipliers 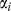
's from the previous iteration and then selects the gene which makes the least change to the dual objective function. The strategy of fixing 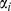
's is likely to compromises the significance evaluation of each gene, as well as the generalization abilities of the resultant SVM classifier.

Advantageously, our algorithms can be directly applied to nonlinear cases by opting for Gaussian RBF kernels. The Gaussian RBF 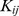, according to Mercer's conditions, is an inner product of 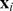 and 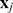 in the feature space: 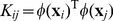
(17)where 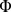 is the function mapping a sample from the input space to the feature space. Thus, for the nonlinear case, the Gaussian kernel matrix is still composed of similarity measurements between training samples.

3. The output of Equation (15) which measures the significance of genes is a real-valued number rather than an integer. This avoids the ties problem [40] which often occurs to count based wrapper methods including the one using the leave-one-out cross validation error as the selection criterion[21].

### Datasets and Data Preprocessing

#### Prostate dataset

The dataset contains, in total, 136 samples of two types which respectively have 77 and 59 cases. Each sample includes expression values of 12600 genes.

#### Colon dataset

The dataset contains the expression values of 2000 genes from 62 tissues, of which 22 are normal and 40 are cancerous.

#### Leukaemia dataset

The dataset was collected from 72 patients. 47 of them were diagnosed with acute acute lymphoblastic leukemia (ALL) and 25 with acute myeloid leukemia (AML). Expression values of 7129 genes were measured.

Both the prostate dataset and the colon dataset were normalized using the following procedure. A microarray dataset with 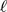 samples and 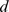 genes was arranged as a matrix of 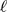 rows and 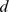 columns. Each row of the matrix was standardized so that the mean and the standard deviation for the row vector are respectively zero and unity. Next, each column of the resultant matrix was standardized to have zero mean and unity standard deviation. No further processing was conducted. All the simulations and comparisons have been performed on the standardized data.

For the leukemia dataset, we applied the pre-processing procedure proposed by Dudoit et al. [41] which consisted of (i) thresholding (floor of 100 and ceiling of 16000), (ii) filtering (exclusion of genes with max/min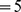 and max-min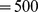 across the samples), (iii) base 10 logarithmic transformation, leaving us with 3571 genes. Next, we applied Fisher's ratio and selected the 1000 top DEGs. For each individual gene, Fisher's ratio assigns it a score using the function 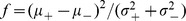 where 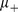 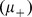 and 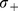 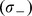 are respectively the mean and the standard deviation across samples from the positive(negative) class. The preprocessing strategy which was also employed by [21] makes possible a fairer comparison between our experiment results and those reported in [21]. All the simulations and comparisons regarding the leukemia dataset have been performed on the preprocessed and pre-selected data.

### Error Rate Estimation Techniques

Various gene selection algorithms are evaluated and compared by the error rate of SVMs. The simplest technique for error estimation is the holdout method which splits the dataset into a training set and a test set. The gene selection algorithm is performed on the training set and sample classification on the test set. However, the holdout method has been highly discouraged for microarray datasets which usually contain a small number of samples. In contrast to researchers who applied gene selection to the entire training set and employed 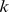-fold cross validation to assess the selected DEGs, Ambroise and McLachlan [42] emphasized to exclude samples used for validation from the gene selection procedure and labelled techniques that follow their recommendation as ''external'' ones. They suggested that external 10-fold cross validation and external B.632+ bootstrap could produce unbiased estimate [42], [43]. Due to the problem of high variance with cross validation techniques when applied to microarray datasets [44], we used the external B.632+ estimator for the comparison of gene selection algorithms.

The B.632+ estimator involves resampling, with replacement, of the original dataset. From a dataset of 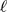 samples denoted as 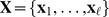, a single sample is randomly drawn and then put back at each time. This process is repeated 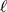 times, leading to a new set which is denoted as 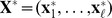. The resampled set 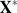 includes, with probability, duplicates of a sample from the original set 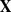. The number of duplicates for a sample included in 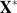 ranges from 0 to 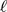. The set of 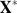 is used for both gene selection and training a SVM. The SVM classifier is then tested on the set of 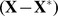. A good error estimator requires the generation of 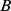 resampled sets which are denoted as 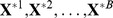 where it was recommended that 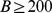. We set 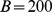 for all our experiments. Meanwhile, for each sample 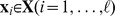, its overall number of occurrences in the 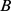 resampled sets is ensured to be 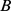, which further reduces the variance.

**Figure 3:**
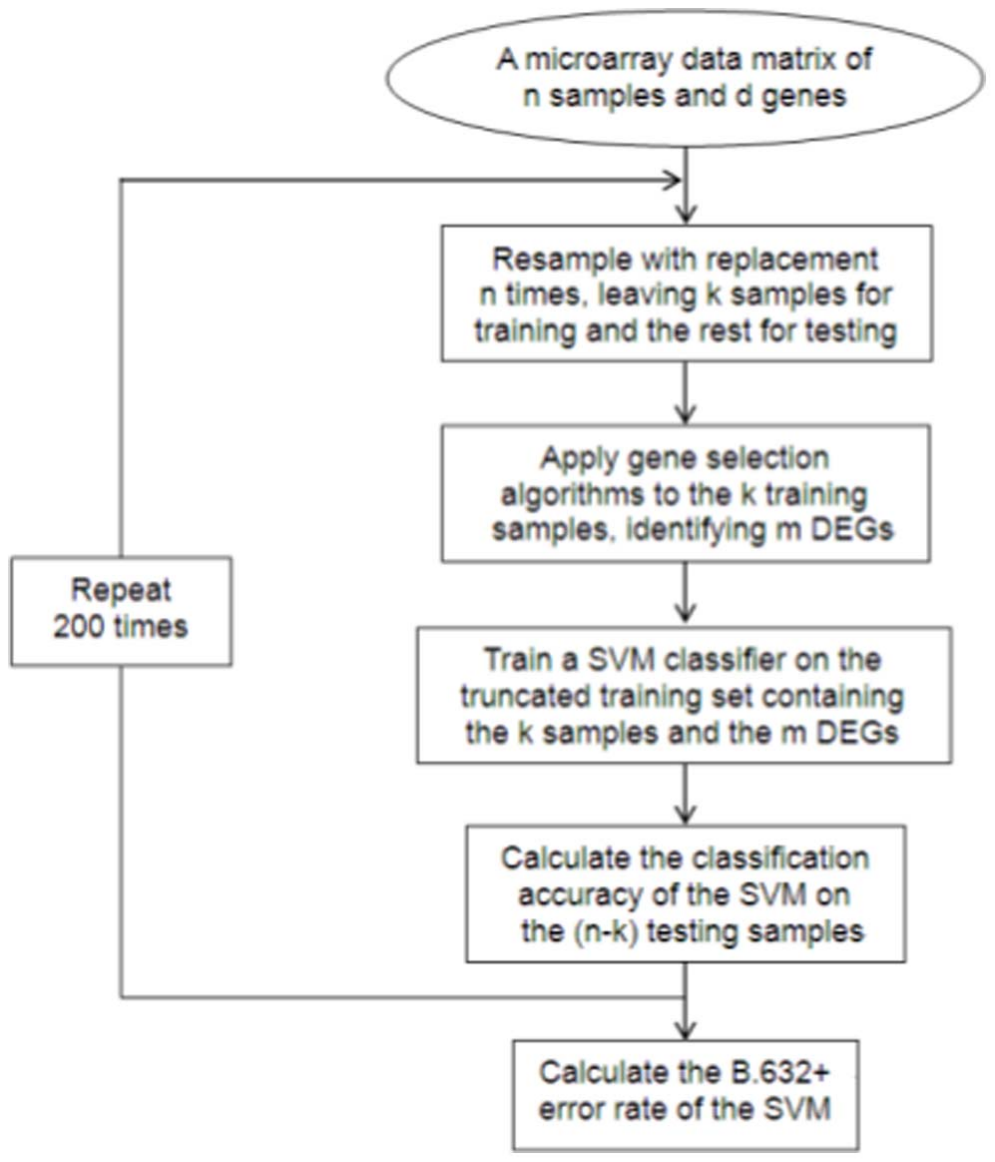
The flow chart for evaluating a gene selection algorithm using the B.632+ technique.

The flow chart for evaluating a gene selection algorithm using the B.632+ technique has been given by Figure 3.

### Gene Selection Algorithms

For the methods of KMSFS and KMGS, both the linear kernel and the Gaussian RBF kernel were tested. This resulted in altogether four algorithms which are referred to as Gaussian KMSFS, Gaussian KMGS, linear KMSFS and linear KMGS. They were compared with two wrapper methods: the leave-one-out calculation sequential forward selection (LOOSFS) which improved the least-squares bound measure [21] by easing the ties problem, and the gradient-based leave-one-out gene selection (GLGS) method [22] which was claimed to outperform the SVM-RFE algorithm [26]. Comparisons were also made to a number of filter methods, including the aforementioned Fisher's ratio [45], Cho's [46] and two other methods of Yang's [47]. We described the ideas of these gene selection algorithms below.

#### Leave-One-Out Calculation Sequential Forward Selection (LOOSFS)

The Leave-One-Out Cross-Validation(LOOCV) error has been generally used for measuring the generalization abilities of SVMs and Least-Squares SVMs (LS-SVMs). The LOOSFS method thus identifies as DEGs those genes which result in a LS-SVM classifier with the minimal Leave-One-Out Cross-Validation(LOOCV) error rate. The beauty of the algorithm consists in the efficient and exact computation of the LOOCV error. To address the ''ties problem'' in which multiple gene subsets achieve the same LOOCV error rate, a further selection criterion is imposed which favors the gene subset with minimal empirical risk.

#### Gradient-Based Leave-One-Out Gene Selection (GLGS)

The starting point of the GLGS method is also the employment of the exact formulation of the LOOCV error for LS-SVMs. The method then utilizes the gradient descent algorithm to seek a diagonal matrix which eventually minimizes the LOOCV error. Genes are ranked according to the absolute values of the diagonal elements of the diagonal matrix.

With Cho's method and Yang's methods, genes are individually ranked. We use the following notations for their descriptions. Each micorarray dataset with 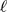 samples and 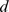 genes is treated as a matrix, denoted as 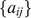, where 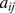 indicates the expression value of gene 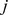 for sample 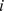. Given a 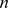-class problem, the average expression value of each class, in terms of gene 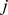, can be computed and denoted as the set of 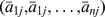. Denote the standard deviation for the set as 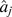. A matrix, denoted as 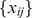 is also introduced, where 
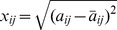
.

#### Cho's Method

The score, denoted as 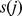, that gene 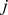 obtains eventually is: 
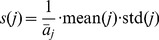
(18)where



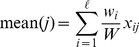
(19)

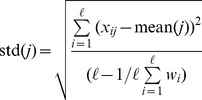
(20)



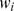
 is the reciprocal of the number of samples that share the same class label as sample 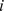 and 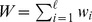. A small value of 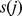 indicates that samples of the 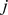-th gene are clustered the centroid of each class.

#### Yang's Methods

The between-class variation with respect to gene 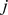, denoted as scatter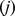, is formulated as: 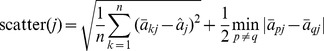
(21)


In order to estimate within-class variations in terms of gene 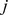, a function 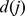 is first introduced: 
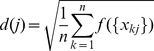
(22)where 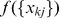 is a function of 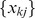 which are composed of the elements from the 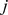-th column of the matrix 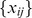 and associated with the 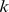-th class. Denote 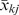 as the mean of of 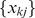. 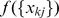 can be either the squared 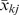 or the mean of 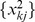, which results in two forms of 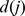 which are referred to as 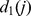 and 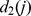 respectively.

Two metrics for measuring within-class variations, denoted as compact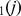 and compact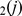 which are derived respectively upon 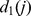 and 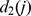, are proposed: 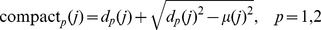
(23)where 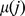 is the mean of the set of 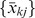 where 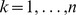.

Eventually, two score functions which decide the ranking of gene 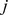, are given:


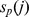
 = 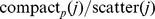, p = 1,2

We refer to these two score functions respectively as Yang's method 1 and Yang's method 2.

Cho's method and Yang's two methods identify as DEGs those genes whose associated 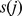 are smaller. For all the gene selection algorithms, it has been emphasized to exclude the test subset each time from the gene selection procedure in order to obtain an unbiased evaluation [42], [43]. The gene selection algorithms terminated when a specific number of DEGs have been identified and we set this number to be 100.

### Parameter Tuning

We employed grid search and Friedman rank sum tests combined with Holm correction to tune the parameters for different algorithms.

#### Grid Search

Among the total 10 gene selection algorithm, both Gaussian KMSFS and Gaussian KMGS require the parameter 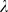 in Equation (13) to be optimized. For the LOOSFS algorithm, the regularization parameter, denoted here as 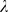 for consistency, has to be tuned. 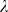 was varied sequentially from 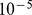 to 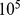 in multiples of 10, which made up a total of 11 different values. With respect to sample classification, the linear SVM was used throughout. Its regularization parameter, denoted as 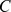, ranged from 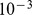 to 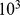 in multiples of 10, which gives 7 different values. Thus, 77 value pairs for 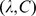 were tested for Gaussian KMSFS, Gaussian KMGS and LOOSFS algorithms, while 7 different values of 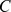 were evaluated for the rest of the 10 gene selection algorithms.

#### Friedman Rank Sum Test with Holm Correction

The Friedman rank sum test is a non-parametric alternative to ANOVA with repeated measures. The test statistic for the Friedman test is a Chi-square with 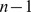 degrees of freedom, where 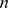 is the number of repeated measures.

We take the algorithm of Gaussian KMSFS as an example to explain how to apply Friedman test for the discovery of optimal values on 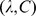. As mentioned previously, 100 DEGs were selected, from each a new classification problem arose. We thus obtained altogether 100 B.632+ error rates for each setting on 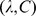. As we tried 77 settings for the parameter pair, 77 groups of classification accuracies were obtained.

Friedman rank sum test was used to detect statistical differences among these 77 groups. The test was based on 100 sets of ranks, with each set corresponding to an individual classification problem. The performances of different parameter settings analyzed are ranked separately for each problem. If we rejected the null-hypothesis stating that all the 77 settings led to equal performance in mean ranking, we employed the Holm post-hoc analysis to identify which setting was significantly better than the rest.

All the gene selection methods were coded in Matlab. The linear SVM was implemented using LIBSVM [48] and the Friedman test with Holm correction was coded in R. The specifications of the computer running the experiments were: Intel core i5-2320 quad-core processor 3.0 GHz, Memory 4 GBytes and the operating system of Windows 7.

## Results

### Results on the Prostate Dataset

#### Optimal Parameter Settings

Using Friedman tests with Holm correction, optimal settings on 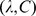 for Gaussian KMGS, Gaussian KMSFS and LOOSFS were found to be 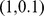, 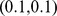 and 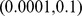 respectively.

GLGS and linear KMSFS shared the optimal setting of 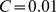. For linear KMGS and all the filter methods which are respectively Fisher's ratio, Cho's method and the two methods of Yang's, the optimal parameter settings were uniformly 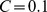.

#### Comparisons against Wrappers

With a minimal error rate of 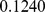 and a mean error rate of 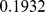, the performance of GLGS was much worse than that of the other 9 methods. Thus its simulation results were not graphically presented.

**Figure 4:**
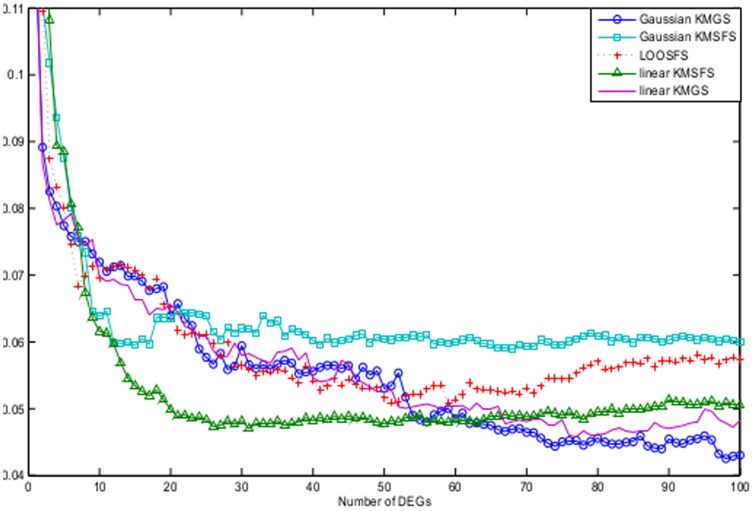
The B.632+ error shown as a function of the number of DEGs for the prostate dataset. The curves depict the performance of the following wrapper methods with their respective optimal parameter settings: Gaussian KMGS with 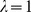 and 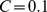; Gaussian KMSFS with 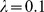 and 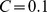; LOOSFS with 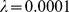 and 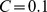; linear KMSFS with 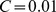; linear KMGS with 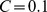. The performance of linear KMSFS was the best when the number of DEGS was between 10 and 60, while Gaussian KMGS outperformed the rest when the number of DEGs increases further to 100. The lowest B.632+ rate was achieved by Gaussian KMGS.

Figure 4 illustrates the the B.632+ error rates as a function of the number of DEGs, for the algorithms of Gaussian KMGS, Gaussian KMSFS, LOOSFS, linear KMSFS and linear KMGS. It can been seen that, when the number of DEGs fell between 10 and around 60, linear KMSFS which is represented by the green solid line dotted with upper triangles, remained the best. As the number of DEGs further increased, Gaussian KMGS outperformed the rest and achieved the lowest B.632+ error rate.

As shown by Figure 4, the classical LOOSFS was outperformed by our algorithms including the Gaussian KMGS, linear KMSFS and linear KMGS. When the number of DEGs ranged between 10 and 20, Gaussian KMSFS also performed better than LOOSFS.

#### Comparisons against Filters

**Figure 5:**
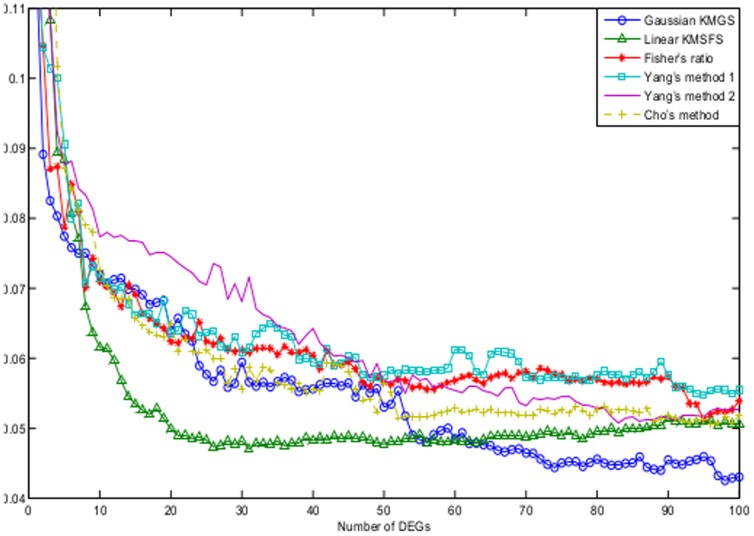
The B.632+ error shown as a function of the number of DEGs for the prostate dataset. The curves are obtained from the following algorithms with their respective optimal parameter settings: Fisher's ratio with 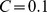; Yang's methods both of which with 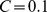; Cho's method with 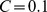; linear KMSFS with 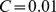; Gaussian KMGS with 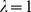 and 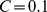. Linear KMSFS and the Gaussian KMGS performed better than the 4 filter methods.

Figure 5 compares linear KMSFS, Gaussian KMGS against the filter methods of Fisher's ratio, Cho's method as well as the two methods of Yang's.

The error rate of linear KMSFS remained noticeably lower than the filter methods when the number of DEGs fell between 10 and 60. When the number of DEGs grew larger, Gaussian KMGS showed better than performance than the four filter methods.

The performance of Gaussian KMGS and linear KMSFS remained competitive to those of the filter methods, respectively between the value ranges of 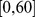 and 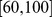 for the number of DEGs.

These comparisons lead to the conclusion that linear KMSFS and Gaussian KMGS are the two best methods for the prostate dataset.

### Results on the Colon Dataset

#### Optimal Parameter Settings

For Gaussian KMGS, Gaussian KMSFS and LOOSFS, the optimal settings on 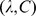 were respectively 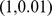, 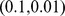 and 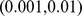.

For the other 3 wrapper methods which are respectively linear KMGS, linear KMSFS, GLGS and the 4 filter methods which are respectively Fisher's ratio, Cho's method and the two methods of Yang's, their optimal parameter settings were found to be 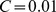.

#### Comparisons against Wrappers

With a minimal error rate of 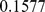 and a mean error rate of 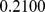, GLGS performed much worse than the other nine methods. Thus again its simulation results were not graphically presented.

**Figure 6:**
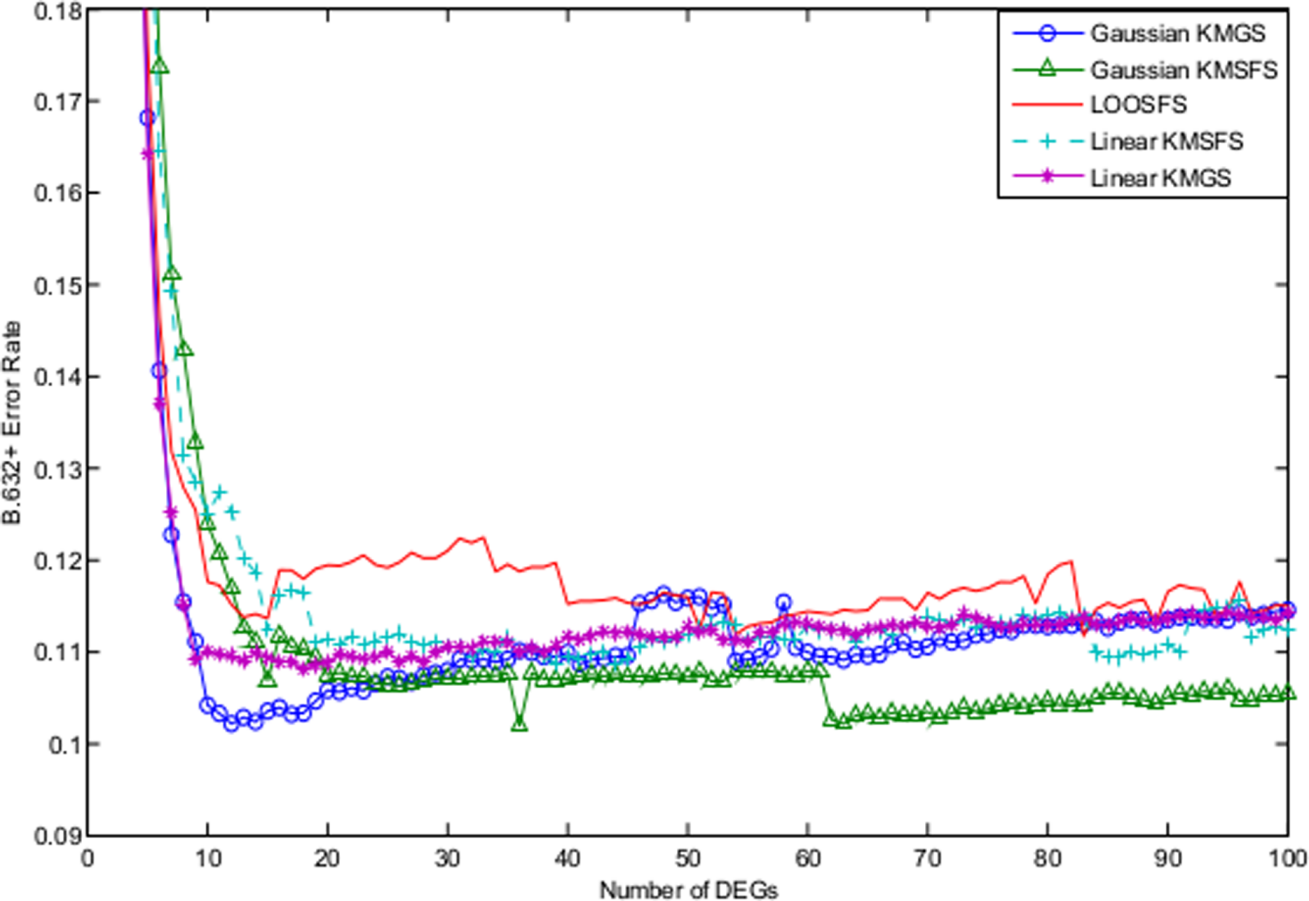
The B.632+ error shown as a function of the number of DEGs for the colon dataset. The curves depict the performance of the following wrapper methods with their respective optimal parameter settings: Gaussian KMGS with 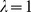 and 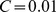; Gaussian KMSFS with 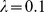 and 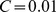; LOOSFS with 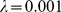 and 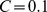; linear KMSFS with 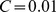; linear KMGS with 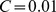. The performance of Gaussian KMSFS was shown to be the best while the performance of the LOOSFS was the worst.

Figure 6 illustrates the the B.632+ error rates of Gaussian KMGS, Gaussian KMSFS, linear KMSFS, linear KMGS and LOOSFS. It can been seen that Gaussian KMSFS demonstrated the best performance while the LOOSFS the worst performance. Gaussian KMGS, linear KMSFS and linear KMGS also performed slightly better than LOOSFS, particularly when the number of DEGs ranged between 15 and 45.

It is interesting to note that Gaussian KMGS, with only 10 DEGs, reached the lowest B.632+ error rate which was approximately 0.10. Also the lowest B.632+ error rate of LOOSFS, which was 0.11 was lower than that reported in [21] which was around 0.15 on the colon data. We reckon it could be due to the employment of different data preprocessing strategies.

#### Comparisons against Filters

**Figure 7:**
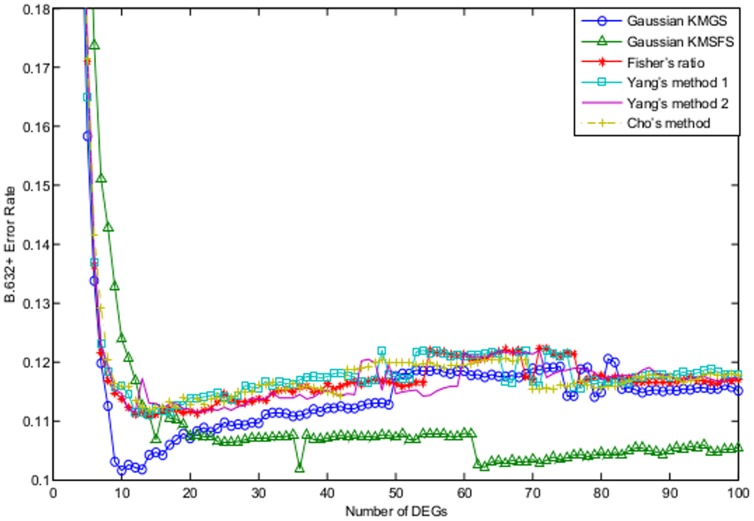
The B.632+ error shown as a function of the number of DEGs for the colon dataset. The curves are obtained from the filter methods among which are Fisher's ratio, Cho's method and Yang's methods with the parameter 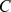 uniformly set at 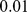. Gaussian KMSFS outperformed all the filter methods noticeably.

Figure 7 compares linear KMSFS and Gaussian KMGS against the filter methods of Fisher's ratio, Cho's method as well as the two methods of Yang's.

Gaussian KMSFS remained better than the 4 filter methods whose performances were comparable between each other. Meanwhile, the error rates of Gaussian KMGS were also lower than those of the filter methods, particularly for a smaller number of selected DEGs.

In conclusion, Gaussian KMSFS and Gaussian KMGS have proved to be the best methods for the colon dataset.

### Results on the Leukemia Dataset

#### Optimal Parameter Settings

For Gaussian KMGS, Gaussian KMSFS and LOOSFS, the optimal setting on the parameter pair of 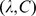 were respectively 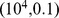, 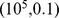 and 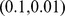.

For GLGS, the optimal setting was found to be 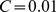. For linear KMGS, linear KMSFS and all the filter methods which are respectively Fisher's ratio, Cho's method and the two methods of Yang's, their optimal parameter settings were uniformly 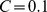.

#### Comparisons against Filters

**Figure 8:**
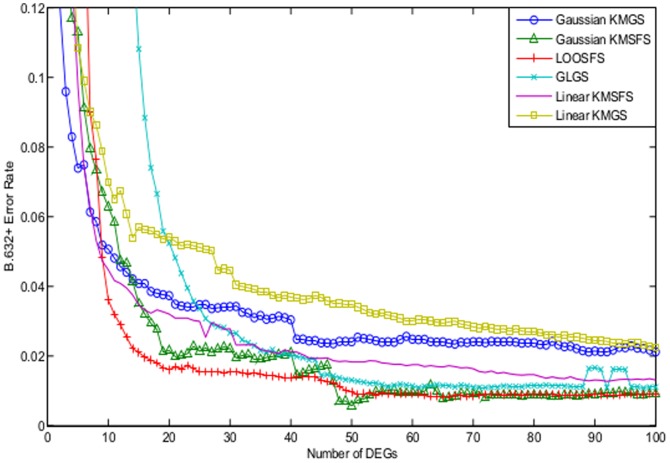
The B.632+ error shown as a function of the number of DEGs for the leukemia dataset. The curves depict the performance of the following wrapper methods with their respective optimal parameter settings: Gaussian KMGS with 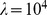 and 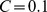; Gaussian KMSFS with 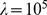 and 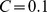; LOOSFS with 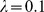 and 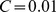; GLGS with 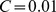; linear KMSFS and linear KMGS with 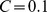. The performance of Gaussian KMSFS remained competitive to that of LOOSFS. Meanwhile, the lowest B.632+ error rate was achieved by Gaussian KMSFS with around 50 selected DEGs.

Figure 8 illustrates the the B.632+ error rates of Gaussian KMGS, Gaussian KMSFS, LOOSFS, GLGS, linear KMSFS and linear KMGS. The performance of LOOSFS depicted by Figure 8 was in fact consistent with that reported in [21]. The performance of Gaussian KMSFS remained competitive to that of LOOSFS. Meanwhile, the lowest B.632+ error rate was achieved by the Gaussian KMSFS with around 50 selected DEGs.

However, Gaussian KMGS, linear KMGS and linear KMSFS failed to perform as well as LOOSFS. We reckon it might be attributable to the preprocessing procedure which resulted in the removal of over 86% of the original 7029 genes, although this viewpoint has to be confirmed with more experiments.

#### Comparisons against Filters

**Figure 9:**
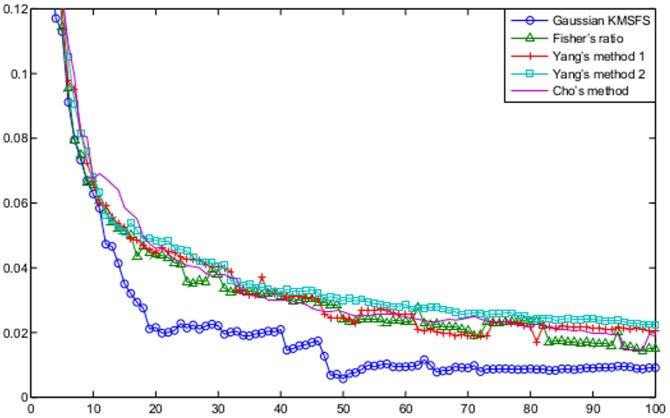
The B.632+ error shown as a function of the number of DEGs for the leukemia dataset. The curves are obtained from the filter methods among which are Fisher′s ratio, Cho′s method and Yang′s methods with 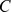 set to be uniformly. Gaussian KMSFS performed better than the 4 filter methods.

Figure 9 further compares the performance of Gaussian KMSFS against the filter methods of Fisher's ratio, Cho's method as well as the two methods of Yang's. It was demonstrated that, the error rates of Gaussian KMSFS remained noticeably low than those of the 4 filter methods throughout.

We regarded the LOOSFS and the Gaussian KMSFS as the two best gene selection algorithms for the leukemia dataset.

## Discussion

### Heatmaps of Differentially Expressed Genes

Due to the employment of B.632+ error estimation technique, each gene selection algorithm was applied to the 200 sets of bootstrap samples as well as the original training set. For each of these 201 sample sets, we selected a sequence of 100 DEGs. This resulted in altogether 201 sets each of which contained 100 DEGs.

We calculated the frequency with which each of the 2000 genes was selected into the 201 sets of DEGs and drew the heatmaps of 50 DEGs that were selected most frequently. For the algorithms of Gaussian KMGS, Gaussian KMSFS and LOOSFS, the outcome of gene selection procedures is influenced by the value setting on the parameter 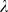 and we used the optimal values reported in the previous section.

**Figure 10:**
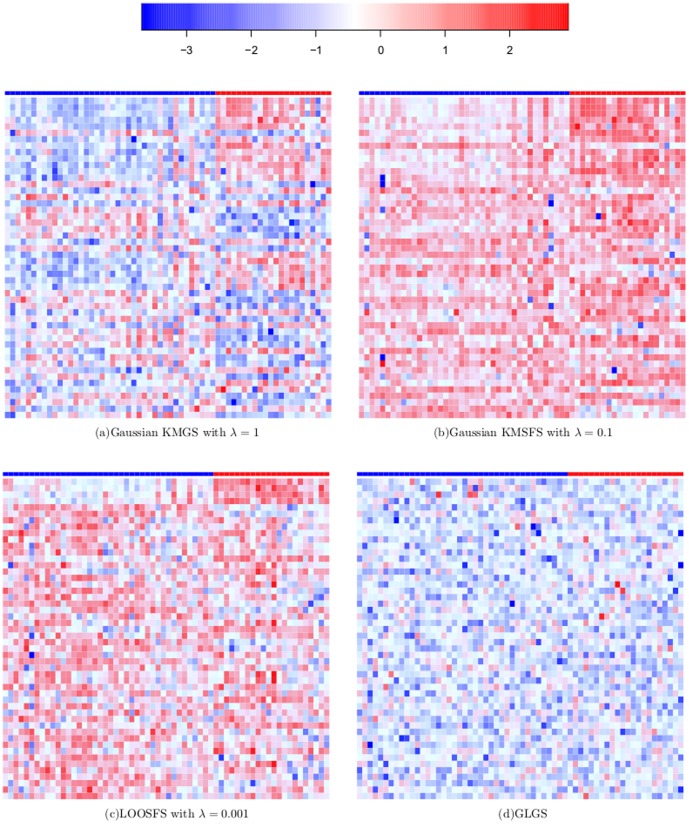
Heatmaps of top 50 DEGs selected most frequently by Gaussian KMGS, Gaussian KMSFS, LOOSFS, GLGS respectively with their optimal parameter settings on 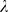.

**Figure 11:**
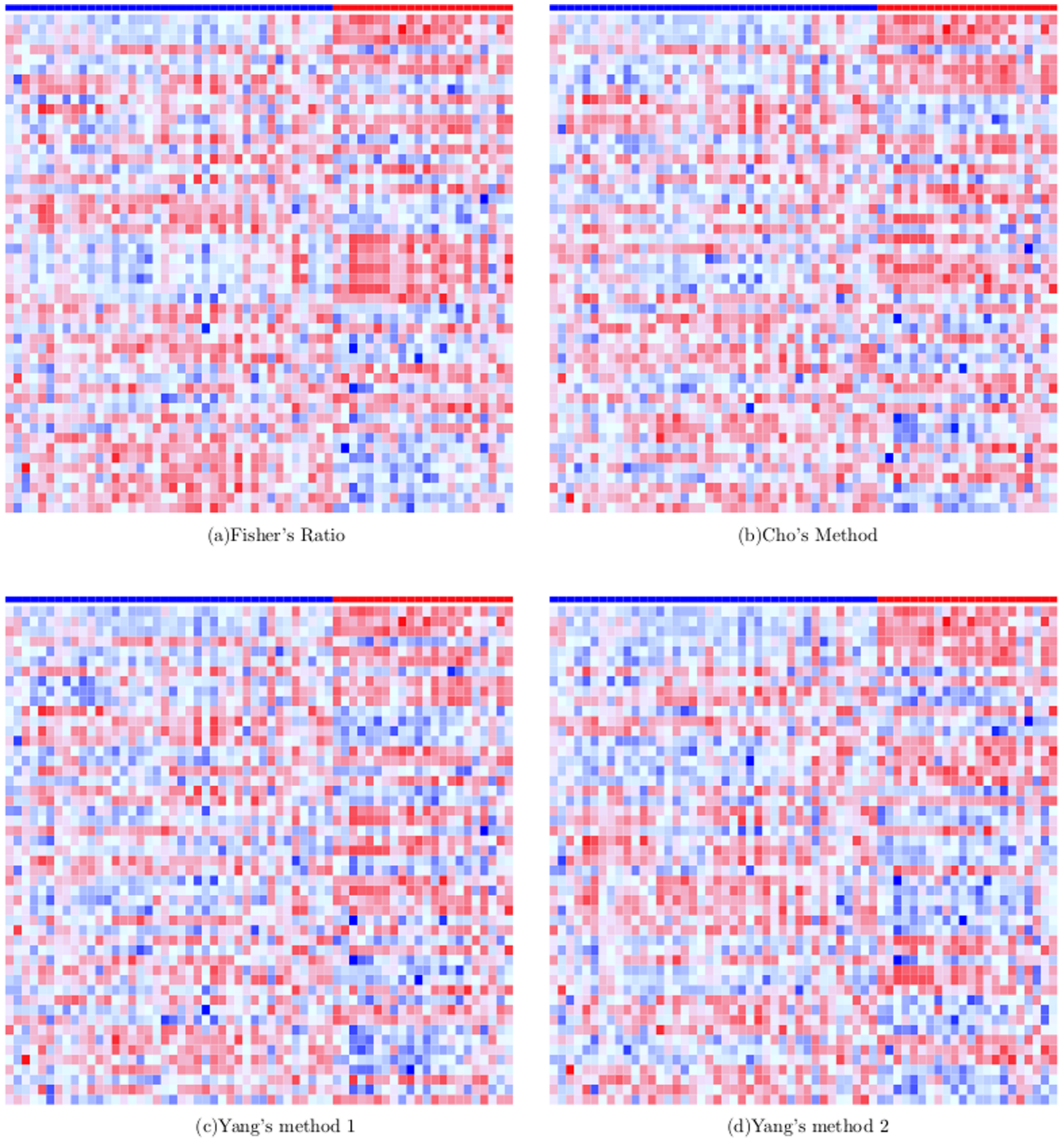
Heatmaps of top 50 DEGs selected most frequently by Fisher's ratio, Cho's methods and Yang's two methods.

**Figure 12:**
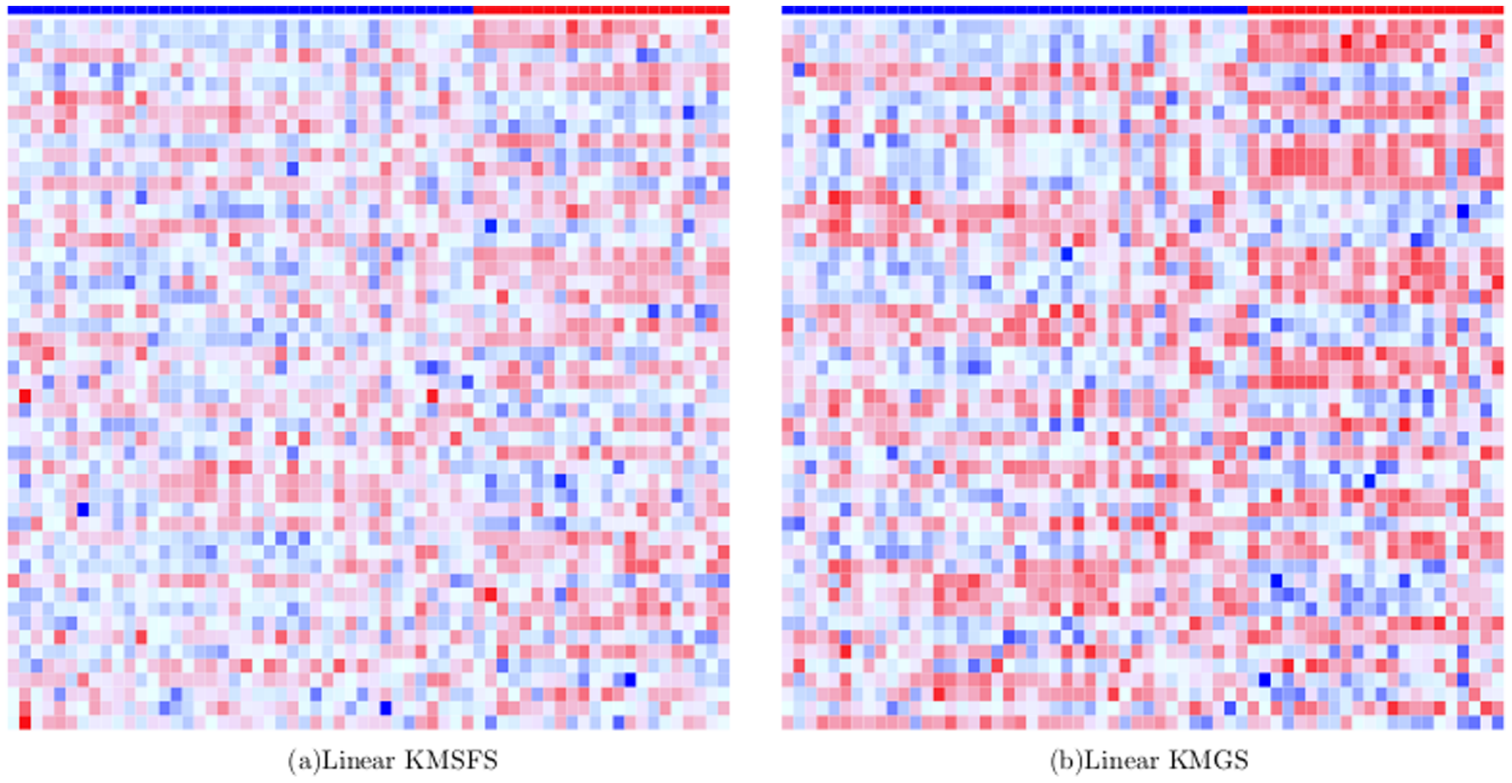
Heatmaps of top 50 DEGs selected most frequently by linear KMSFS and linear KMGS.

Heatmaps for the ten gene selection methods, were shown by Figure 10, Figure 11 and Figure 12. In each heatmap, each column corresponds to a sample and each row is the normalized expression values of a selected DEG across the 62 samples. A grid of each heatmap is colored according to the color key at the top of Figure 10 which maps a normalized expression value to a specific color between blue and red. The class of a sample at each column is indicated by the color bar at the top of each heatmap where blue indicates the cancerous case and red the normal case. Along the downward direction, the 50 DEGs are displayed in descending order of their frequency of occurrence in the 201 sets of selected genes.

It can be seen from Figure 11 that, the filter methods tend to favor ''discriminant features'' whose color forms an obvious contrast between the cancerous population and the normal population at each row. In Figure 10(b) which represents Gaussian KMSFS method, the color of genes at each row is in a pattern of ''occasional dotting of red versus a majority of blue''. The color contrast at each row of Figure 10(b) is less noticeable than Figure 11. Nevertheless, Gaussian KMSFS demonstrated the best prediction accuracies among all the methods, as reported in the previous section.

The second best gene selection algorithm for the colon data is Gaussian KMGS whose selected DEGs have been presented by Figure 10(a). Interestingly, between the two opposing classes, Figure 10(a) exhibited a sharper color contrast than the one exhibited by Figure 10(b).

The above facts suggest that, although filter methods selected genes whose values, in general, differ significantly between opposing classes, our kernel induced algorithms seemed not to hold it as the selection criterion. Instead, our methods endeavored to select genes that could establish a set of ''discriminant samples'' for SVMs. This possibly accounts for their superiority in terms of B.632+ error rates on the colon dataset.

### Parameter Sensitivity Analysis

For linear KMGS and linear KMSFS, their B.632+ error rates are affected by value settings on 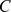. For Gaussian KMGS and Gaussian KMSFS, their B.632+ error rates are influenced by both parameters of 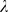 and 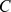.

Using the prostate dataset and the colon dataset, we employed the Friedman rank sum test with Holm correction to study the sensitivity of sample classification to value settings on 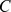 and 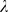 respectively.

#### Sensitivity of Sample Classification to 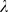


We kept 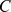 fixed at a specific value and ran Friedman rank sum tests with Holm correction for various choices of 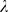. The results were given by Table 3 each row of which reports the score for different values on 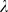 with C fixed at a specific value. The best choice is the one which obtained the lowest score and has been highlighted in bold for each row.

**Table 3:** Scores obtained from Friedman rank sum tests with Holm correction for choices of 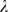 with 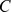 fixed at a specific value at each row.

prostate data												
	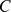 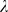	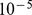	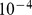	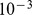	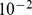	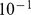	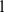	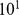	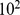	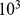	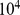	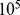
	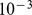	4.095	**4.035**	**4.12**	**4.265**	**5.47**	**4.585**	**5.42**	**6.555**	**8.45**	**9.285**	**9.72**
	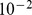	6.165	6.085	5.87	5.11	3.16	**1.78**	**3.15**	**5.67**	**8.2**	**9.92**	**10.89**
	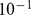	(*) 4.555	4.565	4.43	3.89	2.5	**1.85**	**6.45**	**8.13**	**8.63**	**10**	**11**
Gaussian	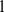	4.66	4.67	4.765	4.16	2.8	**1.92**	**5.455**	**7.85**	**8.72**	**10**	**11**
KMGS	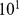	4.95	4.995	4.835	4.38	2.62	**2.28**	**4.98**	**7.56**	**8.4**	**10**	**11**
	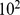	4.875	4.96	4.905	4.32	**2.4**	**2.45**	**5.02**	**7.51**	**8.56**	**10**	**11**
	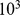	4.82	4.895	4.855	4.25	**2.33**	**2.57**	**5.15**	**7.68**	**8.45**	**10**	**11**
	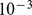	3.82	3.73	**3.535**	**3.965**	**6.12**	**8.545**	**7.07**	**5.835**	**8.44**	**8.25**	**6.69**
	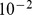	2.74	3.015	3.34	4.065	**2.01**	**6.09**	**7.8**	**9.01**	**7.35**	**9.86**	**10.72**
	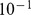	(*) 3.86	4.325	3.96	2.405	**1.14**	**5.31**	**7**	**8.47**	**8.53**	**10**	**11**
Gaussian	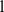	4.08	4.32	4.055	2.385	**1.24**	**4.92**	**7**	**8**	**9**	**10**	**11**
KMSFS	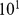	3.965	4.2	3.79	2.165	**1.19**	**5.88**	**6.81**	**8**	**9**	**10**	**11**
	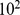	4.005	4.2	3.84	2.155	**1.21**	**6.01**	**6.58**	**8**	**9**	**10**	**11**
	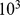	4.005	4.195	3.815	2.085	**1.2**	**6.01**	**6.69**	**8**	**9**	**10**	**11**
colon data												
	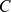 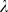	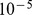	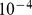	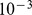	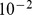	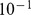	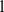	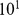	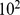	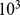	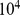	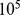
	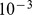	5.01	5.03	5.145	5.345	5.265	7	7.795	8.305	6.905	5.275	**4.925**
	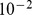	(*) 5.545	5.56	5.465	5.54	5.3	2.715	**2.62**	**4.97**	**8.21**	**9.695**	**10.38**
	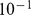	4.815	4.875	4.865	4.94	5.56	3.76	**2.69**	**4.81**	**9.345**	**9.96**	**10.38**
Gaussian	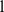	5.32	5.41	5.31	5.455	4.815	3.89	3.31	**2.94**	**9**	**10.225**	**10.325**
KMGS	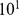	5.32	5.39	5.215	5.44	4.955	3.91	**3.07**	**3.15**	**8.9**	**10.325**	**10.325**
	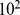	5.295	5.395	5.17	5.385	4.955	4	**3.07**	**3.18**	**8.91**	**10.345**	**10.295**
	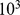	5.31	5.395	5.2	5.39	4.925	4.01	**3.03**	**3.19**	**8.91**	**10.335**	**10.305**
	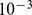	7.47	7.81	7.73	7.525	8.855	**4.435**	**4.435**	**4.435**	**4.435**	**4.435**	**4.435**
	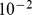	(*) 3.8	3.985	4.185	2.89	**1.39**	**6.02**	**7.02**	**8.42**	**9.595**	**9.335**	**9.36**
	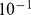	3.23	3.645	4.23	2.77	**1.56**	**5.645**	**7.21**	**8.2**	**9.52**	**10.52**	**9.47**
Gaussian	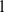	2.735	3.23	3.83	**2.395**	**3.06**	**5.76**	**7.3**	**8.26**	**9.9**	**10.465**	**9.065**
KMSFS	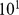	2.655	3.155	3.755	**2.445**	**3.17**	**5.83**	**7.33**	**8.26**	**9.91**	**10.495**	**8.995**
	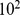	2.61	3.13	3.705	**2.535**	**3.24**	**5.79**	**7.33**	**8.26**	**9.92**	**10.505**	**8.975**
	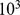	2.6	3.13	3.705	**2.535**	**3.22**	**5.82**	**7.33**	**8.26**	**9.92**	**10.475**	**9.005**

#### Prostate Dataset

We first analyzed the sensitivity of sample selection for Gaussian KMGS. At the row with 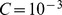, the setting of 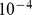 on 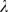 is significantly better than the choices of 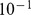,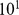,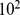, 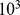,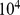,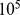 at confidence levels of both 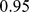 and 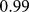. It shows that sample classification is insensitive to 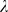 only between 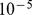 and 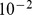 for 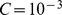. For the row with 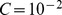, 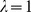 is significantly better than the rest at confidence levels of both 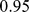 and 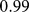.

Since the optimal value setting for 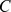 for Gaussian KMGS was found to be 0.1, we have labelled the associated row with an asterisk. It can be seen that, fixing 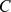 at 0.1, 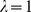 is significantly better than all the rest, except for 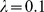, at confidence levels of both 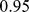 and 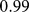. This suggests sample classification is insensitive to choices of 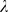 between 0.1 and 1 for 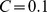. For 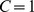, 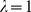 is significantly better than all the rest at confidence levels of 0.95 and 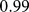. Thus sample classification is insensitive to choices of 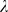 between 0.1 and 1 for 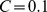.

For the other rows for which 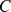 was fixed at 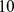, 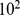 and 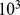 respectively, sample classification remained insensitive to choices of 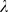 between 0.1 and 1.

We can conclude that B.632+ error rates are insensitive to choices of 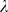 between 0.1 and 1 in terms of Gaussian KMGS.

At the first row for Gaussian KMSFS, we can see that 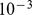 is significantly better than 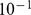 and other larger settings on 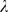. At the next row, 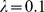 is significantly better than the rest, exclusive of 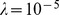, at confidence levels of 0.95. 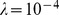 was also excluded at the confidence level of 0.99. The row of 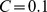 has been labelled with an asterisk, indicating that 0.1 is the optimal choice for 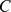 for Gaussian KMSFS. And we can see that 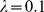 is significantly better than the other settings at confidence levels of both 0.95 and 0.99. This is also the case with the row corresponding to 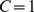.

For the remaining three rows whose 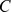 was fixed at respectively 10, 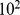 and 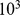, uniformly, 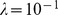 is significantly better than the rest at the confidence level of 0.95.

Thus, for Gaussian KMSFS, B.632+ error rates are sensitive to choice of 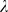, when 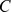 was fixed at 0.1 and 1.

#### Colon Dataset

Similar analysis can be performed for the colon dataset, whose scores for various 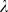 produced by the Friedman tests have been reported at the bottom half of Table 3.

Regarding Gaussian KMGS, we can see that B.632+ error rates are insensitive to choices of 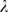 between 10 and 100 when 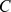 goes from 1 to 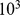 in multiples of 10. For 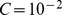 which is its optimal setting, B.632+ rates are insensitive to choices of 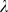 between 0.1 and 1.

In terms of Gaussian KMSFS, we can see that sample classification is sensitive to 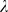 at the rows of 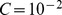 and 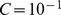. For 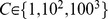, it shows that B.632+ error rates are insensitive to 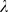 at 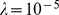 and 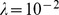. Larger values for 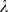 caused severe performance degradation, as suggested by the scores at the bottom right in Table 3.

#### Sensitivity of Sample Classification to 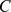


We then kept 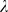 fixed at a specific value and ran Friedman rank sum tests with Holm correction for varied 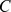
's. The results were given by Table 4 each row of which reports scores for various choices of 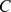 at a specific value setting on 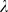. The best choice at each row is the one with the lowest score and has been highlighted in bold.

**Table 4:** Scores obtained from Friedman rank sum tests with Holm correction for choices of 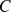 with 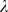 fixed at a specific value at each row.

prostate data														
	Gaussian KMGS							Gaussian KMSFS						
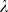 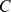	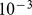	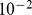	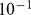	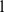	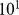	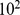	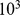	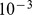	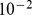	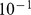	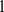	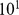	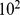	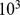
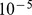	6.99	4.42	**1.26**	**2.99**	**3.96**	**4.12**	**4.26**	**5.57**	**1.58**	**2.28**	**4.32**	**4.58**	**4.805**	**4.865**
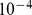	6.99	4.42	**1.26**	**2.99**	**3.97**	**4.115**	**4.255**	**5.195**	**1.57**	**2.485**	**4.365**	**4.625**	**4.85**	**4.91**
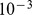	6.99	4.43	**1.26**	**2.98**	**3.94**	**4.13**	**4.27**	**5.27**	**1.57**	**2.29**	**4.425**	**4.705**	**4.84**	**4.9**
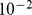	6.99	4.49	**1.26**	**2.95**	**3.96**	**4.1**	**4.25**	**6.99**	**1.52**	**2.355**	**3.905**	**4.35**	**4.415**	**4.465**
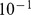	6.99	4.67	**1.22**	**3**	**3.8**	**4.105**	**4.215**	(*)6.99	**1.59**	**1.96**	**3.755**	**4.405**	**4.605**	**4.695**
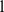	(*)6.99	4.24	**1.12**	**3.115**	**4.075**	**4.15**	**4.31**	**6.98**	**6.02**	**1.44**	**1.89**	**3.98**	**3.8**	**3.89**
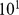	6.99	3.59	**1.45**	**3.325**	**4.105**	**4.265**	**4.275**	**6.93**	**6.07**	**2.52**	**1.88**	**3.34**	**3.525**	**3.735**
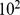	6.99	2.95	**1.4**	**3.61**	**4.27**	**4.315**	**4.465**	**6.93**	**6.07**	**2.49**	**2.18**	**2.94**	**3.635**	**3.755**
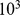	6.99	2.08	**1.65**	**4.03**	**4.31**	**4.465**	**4.475**	**6.89**	**5.12**	**1.92**	**2.52**	**3.44**	**3.965**	**4.145**
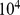	6.93	2.17	**1.63**	**3.495**	**4.295**	**4.625**	**4.855**	**6.63**	**6.11**	**1.8**	**2.58**	**3.32**	**3.72**	**3.84**
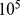	6.83	2.77	**1.68**	**3.2**	**4.11**	**4.615**	**4.795**	**6.19**	**5.95**	**1.6**	**2.375**	**3.67**	**3.985**	**4.23**
	Linear KMGS							Linear KMSFS						
		
NA	6.99	4.64	**1.16**	**3.195**	**3.985**	**3.97**	**4.06**	**4.92**	**1.36**	**3.15**	**4.45**	**4.63**	**4.725**	**4.765**
colon data														
		
	Gaussian KMGS							Gaussian KMSFS						
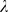 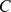	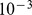	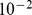	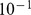	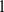	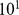	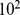	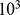	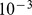	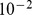	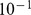	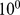	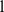	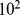	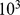
		
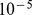	6.195	**1.295**	**2.07**	**4.395**	**4.585**	**4.695**	**4.765**	**6.96**	**1.4**	**2.36**	**4.185**	**4.385**	**4.32**	**4.39**
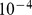	6.195	**1.295**	**2.07**	**4.395**	**4.585**	**4.695**	**4.765**	**6.96**	**1.4**	**2.32**	**4.195**	**4.395**	**4.33**	**4.4**
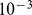	6.185	**1.295**	**2.08**	**4.395**	**4.595**	**4.69**	**4.76**	**6.96**	**1.4**	**2.42**	**4.17**	**4.37**	**4.305**	**4.375**
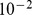	6.235	**1.295**	**2.07**	**4.415**	**4.555**	**4.68**	**4.75**	**6.96**	**1.39**	**2.25**	**4.09**	**4.41**	**4.395**	**4.505**
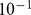	6.255	**1.295**	**2.05**	**4.28**	**4.59**	**4.735**	**4.795**	**6.96**	**1.39**	**2.79**	**4.125**	**4.205**	**4.245**	**4.285**
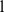	(*)6.96	**1.28**	**1.99**	**3.995**	**4.535**	**4.6**	**4.64**	**6.92**	**1.5**	**1.89**	**3.9**	**4.44**	**4.63**	**4.72**
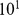	6.915	**1.285**	**2.03**	**3.995**	**4.525**	**4.565**	**4.685**	(*) 5.97	**2.48**	**1.92**	**3.59**	**4.49**	**4.72**	**4.83**
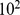	6.855	**1.415**	**2.85**	**3.84**	**4.2**	**4.37**	**4.47**	**5.035**	**2.605**	**1.95**	**4.035**	**4.605**	**4.845**	**4.925**
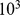	5.735	**1.615**	**2.055**	**4.105**	**4.65**	**4.845**	**4.995**	**4.61**	**2.9**	**1.91**	**4.125**	**4.665**	**4.87**	**4.92**
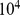	5.555	2.225	**1.93**	**4.025**	**4.665**	**4.73**	**4.87**	**4.62**	**2.86**	**1.81**	**4.215**	**4.645**	**4.87**	**4.98**
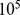	5.52	2.41	**1.99**	**4.055**	**4.545**	**4.685**	**4.795**	**5.325**	**2.685**	**2.1**	**3.95**	**4.56**	**4.64**	**4.74**
	Linear KMGS							Linear KMSFS						
		
NA	6.33	**1.3**	**2.1**	**3.975**	**4.615**	**4.785**	**4.895**	**6.95**	**1.31**	**2.9**	**4.015**	**4.235**	**4.24**	**4.35**

#### Prostate Dataset

For Gaussian KMGS, we can see that B.632+ error rates are sensitive to choices of 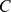. For different 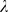
's, 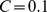 remained the setting that linear SVMs achieved the best performance.

For Gaussian KMSFS, when 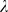 grows from 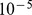 to 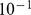, B.632+ error rates remaine sensitive to 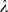 and the best performance was always achieved at 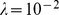. As 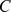 continues to grow larger, B.632+ error rates appear to be insensitive between 0.1 and 1, which is particularly true with the row corresponding to 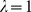.


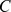
 is the sole parameter for linear KMGS and linear KMSFS. We can also see from Table 4 that, for both algorithms, B.632+ error rates are sensitive to 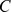.

#### Colon Dataset

Table 4 indicates that, for both Gaussian KMGS and Gaussian KMSFS, the classification performance is insensitive to choices of 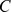 between 0.01 and 0.1, for values of 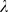 greater than 1. Nevertheless, for smaller values on 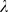 with both algorithms, B.632+ error rates are sensitive to 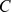.

In term of both linear KMGS and linear KMSFS, B.632+ error rates remain sensitive to 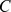, as with the results on the prostate dataset.

## Conclusions

Statistical tests select genes whose expression values differ significantly between the two opposing classes, i.e., the discriminant genes. Samples-based learning machines including SVMs favor genes which results in a set of discriminant samples. The discriminant genes can not be guaranteed to result in a set of discriminant samples. We have shown that the genes leading to discriminant training samples can be detected by applying statistical tests to the kernel matrix.

In addition to the competitive performance demonstrated on the three public microarray datasets, the proposed kernel matrix induced gene selection algorithms offer extra advantages:

1. **Generality**. Our methods are considered applicable to any kernel classifiers, not just SVMs.
2. **Flexibility**. For the implementation of our methods, users can opt for any mercer kernel which can be linear, Gaussian RBF, sigmoid, or polynomials. However, depending on the specific kernel, properly-designed preprocessing steps may be required. For examples, Gaussian RBF kernels require the tuning of the width parameter. For linear kernels, strategies are required to ensure the diagonal elements of the kernel matrix, each of which suggests the similarity between a sample and itself, to be uniformly one.

It is also worth attention that microarray datasets have usually been assumed to present linear problems. However, it is unlikely to be true in the case that the number of DEGs is as few as one. Interestingly, linear problems can be solved by SVMs with nonlinear kernels, while nonlinear problems are hardly solvable with SVMs using linear kernels. A potential solution to the nonlinear problem posed by a small number of DEGs could be the application of nonlinear SVM classifiers. But our method suggested a successful alternative which is the use of the nonlinear Gaussian RBF kernel for the identification of DEGs. We reckon that this effort of instilling ''nonlinearity'' into the identification of DEGs has contributed to the better empirical performance of our methods.

## Supporting Information

Additional File S1 The three microarray datasets (PROSTATE, COLON, and LEU) in MATLAB format which were used in this work.
(ZIP)
